# The Role of Omega-3 Fatty Acids in Developmental Psychopathology: A Systematic Review on Early Psychosis, Autism, and ADHD

**DOI:** 10.3390/ijms18122608

**Published:** 2017-12-04

**Authors:** Carlo Agostoni, Maria Nobile, Valentina Ciappolino, Giuseppe Delvecchio, Alessandra Tesei, Stefano Turolo, Alessandro Crippa, Alessandra Mazzocchi, Carlo A. Altamura, Paolo Brambilla

**Affiliations:** 1 Pediatric Intermediate Care Unit, Department of Clinical Sciences and Community Health, Fondazione IRCCS Ospedale Cà Granda-Ospedale Maggiore Policlinico, University of Milan, 20122 Milan, Italy; alessandra.mazzocchi@unimi.it; 2 SIGENP (Italian Society of Pediatric Gastroenterology, Hepatology, and Nutrition), via Libero Temolo 4 (Torre U8), 20126 Milan, Italy; 3 Child Psychopathology Unit, Scientific Institute, IRCCS Eugenio Medea, via Don Luigi Monza 20, Bosisio Parini, 23842 Lecco, Italy; maria.nobile@bp.lnf.it (M.N.); alessandra.tesei@bp.lnf.it (A.T.); alessandro.crippa@bp.lnf.it (A.C.); 4 Department of Neurosciences and Mental Health, Fondazione IRCCS Ospedale Cà Granda-Ospedale Maggiore Policlinico, University of Milan, 20122 Milan, Italy; valentina.ciappolino@policlinico.mi.it (V.C.); carlo.altamura@unimi.it (C.A.A.); paolo.brambilla1@unimi.it (P.B.); 5 Department of Pathophysiology and Transplantation, Fondazione IRCCS Ospedale Cà Granda-Ospedale Maggiore Policlinico, University of Milan, 20122 Milan, Italy; g.delvecchio@hotmail.it; 6 Pediatric Nephrology & Dialysis, Milano Fondazione IRCCS Cà Grande Ospedale Maggiore Policlinico, University of Milan, 20122 Milan, Italy; stefano.turolo@gmail.com; 7 Department of Psychiatry and Behavioural Neurosciences, University of Texas at Houston, Houston, 77021 TX, USA

**Keywords:** neurodevelopment, omega-3, PUFAs, eicosapentaenoic acid (EPA), docosahexaenoic acid (DHA), autism, attention deficit hyperactivity disorder (ADHD), ultra-high risk for psychosis, first psychotic episode

## Abstract

In this systematic review, we will consider and debate studies that have explored the effects of ω-3 polyunsaturated fatty acids (PUFAs) in three major, and somehow related, developmental psychiatric disorders: Autism, Attention Deficit and Hyperactivity disorder and Psychosis. The impact of ω-3 PUFAs on clinical symptoms and, if possible, brain trajectory in children and adolescents suffering from these illnesses will be reviewed and discussed, considering the biological plausibility of the effects of omega-3 fatty acids, together with their potential perspectives in the field. Heterogeneity in study designs will be discussed in the light of differences in results and interpretation of studies carried out so far.

## 1. Introduction

Mental disorders, despite the growing pharmacological discoveries, will gradually increase in the next decades [1]. Interestingly, among the hypothetical factors that have been reported to increase the incidence of psychopathologies in individuals in developed countries there was the modification of diet habits, mainly due to the transition to more calorically dense ones and to lower level of physical activity [2]. In particular, in recent years, an increased interest has been directed towards the investigation of ω-3 PUFAs in the treatment of different mental disorders [3].

Essential Fatty Acids (EFAs) are obtained from plants or other organisms that possess enzymatic pathways for their synthesis. EFAs are therefore essential dietary nutrients for survival of humans and other mammals because of the absence of the desaturase (Δ)12 and Δ 15 enzymes necessary to insert a double bond at the n ω-6 or -3 position of a fatty acid carbon chain [4]. There are two types of naturally occurring EFAs in the body, the ω-6 series derived from cis-linoleic acid (LA, 18:2) and the ω-3 series derived from α-linolenic acid (ALA, 18:3). Once obtained from the diet, LA and ALA are further metabolized in the liver by the enzymes Δ6 and Δ5 desaturases to generate long-chain PUFAs (LCPUFAs) as arachidonic acid (AA, 20:4, ω-6), eicosapentaenoic acid (EPA, 20:5, ω-3) and docosahexaenoic acid (DHA, 22:6, ω-3). The enzymatic metabolic products of 20 carbon LCPUFAs are called eicosanoids and they include prostaglandins, thromboxanes, and leukotrienes: in general, AA-derived eicosanoids have mainly pro-inflammatory properties, whereas EPA derived eicosanoids are rather less inflammatory. Furthermore, recent studies have identified a novel group of mediators termed E- and D-series resolvins formed from EPA and DHA, respectively. Together with neuroprotectin D1 formed from DHA via several reactions, these mediators appear to exert strong inflammation resolving effects [5]. This suggests that LCPUFAs form precursors to both pro- and anti-inflammatory molecules, and the balance between these mutually antagonistic compounds could determine the final outcome of the disease process [6].

Moreover, LCPUFAs are incorporated into membrane phospholipids and the incorporation of DHA takes place in uniquely high levels in the central nervous system [7]. This accumulation in the brain starts during the brain growth spurt in the intrauterine stage and continues up to two years of age, then maintaining high levels throughout the life span [8]. The intrauterine LCPUFAs supply occurs via transfer of non-esterified PUFA mainly derived from the maternal circulation across the placenta [9]. Post-natal accumulation of LCPUFAs in infant tissues is supported by maternal transfer of LCPUFAs through breastmilk, and blood levels of LCPUFAs in breast-fed infants remain higher than maternal levels for some time postnatally [10]. With respect to the functional effects of LCPUFAs supplementation in infancy, the most accepted developmental effect is an increased rate of visual acuity development, which seems to be explained solely by DHA [11]. Once high levels of DHA are achieved in the brain, these are maintained during later life, nevertheless this presumably still depends on an optimal dietary supply, as dietary intake of DHA from fish in adults has been shown to be the dominant determinant of DHA levels in various lipid pools [12]. In agreement with these observations, the implementation of LCPUFAs in psychiatric disorders stemmed from the fact that the brain is composed, in large part, of the ω-3 PUFAs, which have been shown to have neuroprotective properties, especially in producing modification of the synaptic membrane [13]. Indeed, they modulate brain cell signaling, including monoamine regulation, and are involved in the modification of receptor properties or the activation of signal transduction by receptors [14,15,16,17], which may explain their role in the onset of certain psychiatric diseases [18,19]. 

Omega-3 PUFAs deficiencies have been reported in people with a wide range of mental disorders, including attention deficit hyperactivity disorder (ADHD), depression, schizophrenia (SCZ), and Autism Spectrum Disorder (ASD) [13,16,20,21,22,23,24]. In particular, previous studies observed significant abnormalities in the fatty acid composition of peripheral tissues from drug-naïve first-episode patients with schizophrenia (SCZ) relative to normal controls, including deficits in ω-3 and ω-6 PUFAs, which are partially normalized by chronic antipsychotic treatment [24]. Similarly, medication-naïve first-episode psychotic patients also showed erythrocyte DHA deficits compared with healthy controls [25,26], and a recent meta-analysis of 18 case-control studies reported significant DHA deficits in patients with SCZ [27], the presence of reduced concentrations of ω-3 PUFAs has been reported in children with ASD [28]. Further confirming the key role played by ω-3 PUFAs in neurodevelopment, data from observational studies consistently indicated lower ω-3 PUFAs blood levels (especially DHA) in children with ADHD compared with their typically developing peers [29,30]. Several studies accordingly reported a significant negative relationship between blood ω-3 PUFAs status and ADHD symptoms: low blood levels of DHA and EPA have been shown to be associated with ADHD symptoms such as inattention and hyperactivity [31,32,33]. Based on this evidence, an increasing number of intervention studies have been conducted to evaluate the efficacy of ω-3 PUFAs supplementation for the treatment of children with ADHD.

In this context, the aim of this systematic review is to provide an updated account of the evidence of the efficacy and safety exerted by ω-3 PUFAs supplementation in ASD, ADHD and early psychotic symptoms (ultra-high risk of psychosis and first psychotic episode) to check the hypothesis that ω-3 PUFAs might be useful either sole or in augmentation as a potential therapy for these disabling psychiatric disorders.

## 2. Results

### 2.1. Omega-3 in Psychosis

The worldwide prevalence of psychosis has been estimated to be approximately 1% [12]. Notably, it has been reported that this mental disorder leads to long term disability [34] by disrupting social and family relationships, which in turn determines severe educational and occupational impairment, lost productivity, unemployment, physical illness, and premature mortality [35]. The main symptoms experienced by psychotic patients are auditory hallucinations and delusional ideation [36]. Psychotic illnesses are usually preceded by a prodromal period, lasting one to three years [37], which is characterized by a range of non-specific behavioral and psychological symptoms, functional deterioration, and by both attenuated positive symptoms and brief limited intermittent psychotic symptoms [38]. Prospective studies of individuals who later develop psychosis have given the opportunity to analyze the potential risk factors, such as heritability, linked to this mental disorder. Over the past decade, the term “ultra-high-risk” (UHR) identifies adolescents and young adults who are at increased risk of developing full blown psychotic symptoms; among these subjects about 22% to 40% shows transition to overt disease within 12 months [39,40,41]. Therefore, interventions that prevent or delay transition to psychosis from the prodromal phase may be clinically and economically important [42]. It has been suggested that the treatment with ω-3 PUFAs have some potential beneficial effects on psychotic symptoms. This evidence, along with the findings showing lower levels of ω-3 and ω-6 PUFAs in patients with SCZ compared to healthy controls [24,25,26,27,43], has allowed to put forward the “membrane phospholipid hypothesis” of SCZ [44,45]. Similarly, many epidemiological or observational studies also suggested that greater dietary intake of fish or ω-3 PUFAs is linked to a general reduced risk of psychosis [46,47,48,49]. In this paragraph, we summarized all randomized clinical trial studies (RCTs) with the final aim of providing a clearer picture of the impact of EPA and DHA in UHR subjects and in first episode psychosis patients.

To date, only five RCTs explored the role of ω-3 PUFAs supplementation in UHR patients or in first episode of psychosis (Table 1).

#### 2.1.1. Ultra-high-risk Patients

The study with the larger sample is a multicenter RCT study carried out by McGorry et al. [50] on 304 subjects meeting the criteria for being “at risk” to develop psychosis. They were randomized to daily dose of 1.4 g of ω-3 PUFAs (EPA 840 mg + DHA 560 mg) or placebo (paraffin oil), without the add-on therapy with neuroleptics or mood stabilizers but with the use of antidepressants and benzodiazepines. In addition, 20 or fewer sessions of cognitive behavioral case management (CBCM) were administered over the 6-months study period with an additional follow up of six months. The primary outcome was transition to psychosis status at 6 months. The authors showed no significant differences between the transition rates to psychosis and outcomes between the groups, with low transition rates and overall symptomatic functional improvement in both arms. These results could be probably due to the efficacy of CBCM and antidepressants that both groups have received, which may have hidden the effectiveness of ω-3 PUFAs. In contrast, Amminger et al. (2010) [46] in a 12-week RCT reported the efficacy of ω-3 PUFAs pills (containing 700 mg of eicosapentaenoic acid and 480 mg of docosahexaenoic acid) versus placebo (coconut oil + Vit E 7.6 mg) in 81 individuals at UHR for psychosis in addition to antidepressants and psychological treatments. Interestingly, no difference for side effects were observed between the ω-3 PUFAs and placebo groups. The positive effect of ω-3 PUFAs treatment was further confirmed by the same research group in three other studies employing the same or part of the sample [51,52,53], showing reduced risk of progression to psychosis over a long follow-up period (median 6.7 years). Amminger et al. [52] also reported that the UHR subjects with higher levels of erythrocyte membrane ALA may specifically benefit from ω-3 PUFAs treatment. Interestingly, Smesny et al. [53] demonstrated that ω-3 PUFAs supplementation may act by normalizing intracellular phospholipase A2 activity and Δ-6-desaturase-mediated metabolism of ω-3 and ω-6 PUFAs.

#### 2.1.2. First Episode Psychosis

We identified two RCT studies reporting contrasting results. Pawelczy [54] demonstrated a significant difference between ω-3 PUFAs and placebo in first episode of psychosis patients, whereas Berger [55] found no significant difference between the same groups. These discordant results may be due to different doses of ω-3 PUFAs, administration periods (6 vs. 3 months), type of ω-3 PUFAs (EPA + DHA vs. EPA) or patients’ clinical characteristics. Specifically, Pawelczyk found after a period of six months a significant symptoms improvement in ω-3 PUFAs group (2.2 g/day: EPA 1320 mg + DHA 880 mg) vs. placebo in a sample of 71 patients suffering from first-episode psychosis, as add-on treatment to antipsychotics. In contrast, Berger [55] observed a higher response rate to antipsychotic drugs (risperidone, olanzapine or quetiapine) at Week 6 for first-episode psychosis patients supplemented with ethyl-EPA (E-EPA at 2 g/day) in comparison to placebo, which disappeared at Week 12. Additionally, they found that patients on E-EPA compared to those on placebo: (a) needed 20% less antipsychotic medication; and (b) had less extrapyramidal, gastrointestinal and sexual side effects. Therefore, these results suggest that E-EPA accelerate treatment response and ameliorate the tolerability of antipsychotics, particularly during the first 4–6 weeks of treatment. Interestingly, the same research group in 2008 [56] by means of proton magnetic resonance spectroscopy showed that 12-weeks of E-EPA augmentation in first-episode psychosis patients increased the levels of glutathione in temporal lobes, which is involved in oxidative stress [57] and/or apoptotic activity [58], and the ratio of glutamate/glutamine in the left hippocampus. These changes could partially explain the neuroprotective effects of E-EPA and may potentially ultimately help in preventing neurodegenerative processes taking places in psychotic disorders. 

Interestingly, although the evidence reporting the effectiveness of ω-3 PUFAs in psychosis is still on its infancy, several meta-analyses conducted in patients affected by SCZ [59,60,61,62,63], at risk to develop psychosis [42,64,65,66], or at first episode of psychosis [67,68,69] further support the abovementioned results. Future studies with larger and more homogeneous sample are needed to elucidate the efficacy of ω-3 PUFAs in UHR subjects or in first episode of psychosis.

### 2.2. Omega-3 and Autism

Autism spectrum disorders (ASD) refers to a group of conditions related to neurodevelopment including impaired social behavior, restricted communication, repetitive activities and limited interests. The etiology is primarily linked to genetics but also environmental factors may play a relevant role.

The World Health Organization (WHO) updated the prevalence estimating that worldwide 1 in 160 children has an ASD. The disorder is evident in early childhood and tends to persist into adolescence and adulthood.

Despite progress in medically treating ASD, there is an increasing interest in alternative treatments including melatonin, vitamins, a gluten- and casein-free diet, and the use of ω-3 PUFAs, although the biological plausibility is quite different among these alternatives. Indeed, ω-3 PUFAs and their metabolic products offer a solid basis, since they have been implicated in ASD via their roles in brain structure and function, neurotransmission, cell membrane structure and microdomain organization, inflammation, immunity and oxidative stress. There is accumulating data to support that EPA and DHA are important for brain structure and function and have been advocated for the treatment of multiple neurodevelopmental disorders including mood disorders, schizophrenia, ADHD, and ASD.

Abnormality in blood levels of ω-3 PUFAs has been reported in psychiatric disorders including, but not limited to, ADHD and ASD [23,24,25]. In particular, compared with typically developed, ASD populations had a lower DHA, EPA and ARA and higher total ω-6 PUFA to ω-3 PUFAs ratio [70]. Elevated levels of several peripheral pro-inflammatory cytokines and nuclear factor Kappa B (NF-κB, a transcription factor involved in inflammatory signaling pathways) has been reported in children with ASD [70].

In this paragraph, we provide an update regarding the effects of ω-3 PUFAs supplementation on symptoms of ASD in children. We included RCTs reporting outcome measures as core symptoms of ASD (including social interaction, communication and repetitive restrictive behaviors or interests) and symptoms or behaviors associated with ASD (e.g., hyperactivity).

We identified three RCT studies (Table 1). The first RCT analyzed [71] is an internet-based RCT (IB-RCT) conducted to examine the feasibility of a novel internet-based design in order to evaluate the safety and efficacy of ω-3 PUFAs in ASD. Children (*n* = 57) affected by ASD between the age of five and eight were randomly assigned to six weeks of treatment (*n* = 29) with 1.3 g of ω-3 PUFAs (and 1.1 g of DHA + EPA) or an identical placebo (*n* = 28). 

The primary outcome measure was defined a priori as the change in hyperactivity on the Aberrant Behavior Checklist (ABC-H, parent and teacher) over the six-week treatment period: children in the treatment group did have greater mean improvements in hyperactivity compared to the placebo group (a 5.3 point reduction in the ABC-H in the ω-3 PUFAs group vs. 3.4 in the placebo group), but this difference was not significant. Concerning the secondary outcome measures, this study found statistically significant improvements in the ω-3 PUFAs group in two secondary outcome measures, the stereotypy and lethargy subscales of the ABC.

The second RCT [72] as conducted by Voigt and presented different conclusions. In particular, 48 children 3–10 years of age with ASD were randomized to receive a dietary DHA supplementation of 200 mg/day for six months (*n* = 24) or placebo (*n* = 24).

The authors did not show any improvement in core symptoms of autism or a broad range of associated developmental and behavioral difficulties. They only found a favorable change in functional communication reported by teachers in children with autism who received DHA supplementation but further investigations are required.

The third study [73] is a 16-week RCT that evaluated the efficacy of supplementation with large doses of ARA added to DHA (*n* = 7) (240 mg of ARA-enriched triglyceride containing 40 mg/capsule each of ARA and DHA, and 0.16 mg/capsule of astaxanthin) or placebo (*n* = 6) in 13 participants in the age range 6–28 years who had autistic disorder. The results suggest that supplementation with larger ARA doses added to DHA improves impaired social interaction in individuals with ASD.

The treatment effect sizes for ABC social withdrawal (treatment group: 0.88 vs. placebo: 0.54) and Social Responsiveness Scale (SRS) communication (treatment group: 0.87 vs. placebo: 0.44) subscales were more favorable for the treatment group than for the placebo group.

In conclusion, these findings reported a small but not significant benefit of ω-3 PUFAs supplementation in children with ASD. Data in this field are few and the sample size is often too limited. This is in agreement with recent systematic reviews and meta-analysis [70,74] concluding that, based on the current evidence, ω-3 PUFAs supplementation cannot be recommended as an alternative to support behavioral therapies for ASD children, but could be used to complement other therapies. Large high-quality RCTs are still required to further clarify the role of ω-3 PUFAs on functional outcomes in this population and to draw conclusions.

### 2.3. Omega-3 in ADHD

The association between ADHD symptoms and low blood levels of ω-3 PUFAs represents a consistent finding among observational studies. Driven by this evidence, several intervention studies have been carried out in the last 15 years to evaluate possible beneficial effects of ω-3 PUFAs in the treatment of behavioral and cognitive symptoms of children with ADHD. Our aim was to review current evidence from RCTs investigating the efficacy of ω-3 PUFAs supplementation on ADHD symptomatology. The main characteristics of the 25 RCTs on ADHD included in this review are shown in Table 1. The majority of studies (*n* = 17) employed ω-3 PUFAs as monotherapy, whereas eight studies used ω-3 PUFAs as an adjunctive therapy to methylphenidate (MPH) or atomoxetine (ATMX). Sample age ranged from 4 [75] to 18 [76] years. Among studies, different ω-3 PUFAs compositions were used as supplementation: EPA or DHA alone [72,77], EPA with DHA [75,78,79,80,81,82,83,84,85,86,87], EPA combined with DHA and other ω-3 PUFAs or ω-3 PUFAs [76,88,89,90,91,92,93,94], and, finally, short chain ω 3-PUFAs [95,96]. Two studies declared to have supplemented with ω-3 PUFAs alone [97] or in combination with ω-6 PUFA [98], without specifying which type of fatty acid. 

Two RCTs were performed supplementing children with ADHD with either DHA or EPA alone, as add-on therapy. The first study was carried out by Voigt et al. [99], in which placebo or 345 mg/day of DHA were administered to children with ADHD for four months. In the second study [77], ADHD children were divided between those receiving EPA (100–400 mg/day), those receiving zinc supplement and a placebo group. For both studies, no significant difference between groups was found on any cognitive or behavioral outcome. However, Salehi [77] performed a subgroup analysis only on children with attention-deficit disorder subtype, revealing a better clinical response on Conners’ Rating Scales over the eight weeks of treatment in EPA group than zinc group.

With regard to RCTs exploring the effect of combined EPA and DHA at different dose levels, 11 studies have been identified. Among them, six reported some improvement in ADHD symptoms, whereas the other five studies did not find any significant ameliorating effect.

In a recent RCT [75], 50 children with ADHD were randomized to receive ATMX or ATMX and ω-3 PUFAs (180 mg EPA, 120 mg DHA) for four months. Supplementation with ω-3 PUFAs brought a reduction of symptoms assessed via Conners’ Parent Rating Scale (CPRS), but at a non-significant level. However, children with combined type of ADHD showed a statistically significant improvement over four months compared to the other two types (inattentive and hyperactive). Behdani et al. [78] investigated whether ω-3 PUFAs treatment (360 mg/day EPA, 240 mg/day DHA) could enhance therapeutic effects of methylphenidate (MPH) in 69 children with ADHD. After eight weeks of treatment, both the study group—supplemented with omega-3 plus MPH—and the control group—supplemented with placebo plus MPH—showed significant reductions in ADHD rating scale (ADHD-RS) scores, but there was no significant difference between two groups. Similar results were observed by Bélanger and colleagues [80] in their cross-over RCT, in which they administered as monotherapy either ω-3 PUFAs (20–25 mg/kg/day EPA, 8.5–10.5 mg/kg/day DHA) or placebo to 26 children for eight weeks. They observed a statistically significant amelioration in several CPRS subscales in both groups, however, treatment group showed greater but not statistically significant improvement in comparison with placebo group. Milte et al. [84,85] decided to compare, in the context of a 12-month randomized, controlled, three-way crossover trial, EPA- versus DHA-rich supplements, using LA-rich oil as placebo. Eighty-seven children with ADHD symptoms were divided in three groups, each receiving DHA-rich oil, EPA-rich oil and LA-rich oil for four months each. Results showed no significant treatment effects on cognitive/behavioral variables; however, significant associations were found between increased blood levels of DHA + EPA and improved literacy, attention and parent-rated behavior on CPRS.

Focusing on the six trials of DHA + EPA supplementation reporting improvement on ADHD symptoms, they all choose a minimum duration of 12 weeks and a minimum sample size of 40. Recently, Bos et al. [79] found a specific effect of DHA + EPA (administered in equal doses of 650 mg) on parent-rated attention problems over placebo on 40 children with ADHD following 16 weeks of treatment. This result is in agreement with a previous RCT of Gustafsson et al. [81], where supplementation with 500 mg/day of EPA and 2.7 mg/day of DHA improved inattentive problems but not hyperactive behavior. Kean et al. [82] also reported improvement on several CPRS subscales in children displaying high levels of inattention and not in those with high hyperactivity. Results in favor of a treatment effect on other core symptoms of ADHD comes from Manor et al. [83], whose trial—conducted on 147 ADHD children—showed a significant reduction of parent-rated restlessness and impulsiveness as rated by parents in the EPA + DHA group compared to placebo. Concerning neuropsychological functioning, three RCTs using higher doses of EPA than DHA [82,86,87] found a treatment effect on visual sustained attention performance, delayed memory and working memory function. Another RCT, conducted by Sinn et al. [94] used a slightly different supplement composition i.e., the addition of GLA (γ-linolenic acid, omega-6) to EPA and DHA, and also found a significant improvement in the treatment group compared to placebo on a cognitive measure, which is the ability to switch and control attention. This latter study belongs to that group of RCTs using EPA, DHA and other ω-3 PUFAs or ω-6 PUFAs as supplementation; beside it, four other studies have shown some beneficial effects on ADHD symptoms, whereas three studies did not. Sinn et al. [93], using the same sample and fatty acid composition of the above-mentioned study, observed a significant improvement in the PUFA group than placebo on all core ADHD symptoms—inattention, hyperactivity/impulsivity—as rated by parents. Stevens et al. [92] chose to administer to treatment group (*n* = 25) higher doses of DHA than EPA (480 and 80 mg, respectively) with the addition of AA (40 mg) and GLA (96 mg) compared with a placebo (*n* = 25). For attention problems rated by teachers and for conduct problems rated by parents, PUFA showed a small but significant effect over placebo whereas no treatment effect was found on neuropsychological measures (continuous performance test and other tests on short-term memory, processing speed, auditory processing and visual processing). Hariri et al. [90], in a sample of 103 children with ADHD under stimulant medication, compared the efficacy of 635 mg/day of EPA, 195 mg/day DHA and 100 mg/day of other omega-3 fatty acids versus placebo and observed a significant improvement on Conners’ Abbreviated Questionnaire (ASQ-P) after eight weeks for supplemented patients but not for those in the placebo group. Johnson et al. [76] randomized 64 ADHD children to receive for 12 weeks either 558 mg/day EPA, 174 mg/day DHA and 60 mg/day GLA or placebo. Despite finding significant greater improvement on symptom severity and functional impairment (measured by Clinical Global Impression severity scale) in the supplemented than in the placebo group, authors considered their RCT an “essentially negative study”, as only 26% of supplemented children responded to treatment with more than 25% improvement of ADHD symptoms on ADHD-RS.

Two recent studies [87,88] tested the efficacy of DHA + EPA + ω-6 PUFA with MPH over MPH monotherapy and came to similar negative results. Barragán et al. [89] used a fatty acid supplementation that contained a higher ratio of EPA (558 mg/day) than DHA (174 mg/day) and some GLA (60 mg/day); on the contrary, Assareh et al. [88] used a higher ratio of DHA (241 mg/day) than EPA (33 mg/day), together with 180 mg/day ω-6 PUFA. Barragán et al. [89] observed a decreasing in ADHD symptoms in all treatment arms, with no superior effect of PUFA + MPH as compared to MPH monotherapy on ADHD symptoms. However, the combination of PUFA + MPH lowered the frequency of adverse events than MPH alone and appeared to be more effective than PUFA alone for hyperactivity/impulsivity, whereas results on inattention were similar. Even Assareh, despite finding in their RCT a significant improvement of core ADHD symptoms within both treatment and control group after 10 weeks, reported no statistical superiority of MPH + PUFA compared with MPH + placebo. Lastly, negative results come also from Matsudaira and coworkers’ study [91], in which they did not find any significant ameliorating effect of EPA + DHA + GLA supplementation (respectively, 558, 174 and 60 mg/day) compared to placebo on ADHD symptoms over 12 weeks.

As mentioned above, two RCTs have supplemented with ω-3 PUFAs alone [97] or in combination with ω-6 [98], without specifying which type of fatty acid. Dashti et al. [97] divided 85 children with ADHD into three groups: those receiving ω-3 PUFAs, those receiving MPH and those receiving placebo. They reported a statistically significant improvement of ADHD symptoms for children in the ω-3 PUFAs group or in the MPH group but not for children in the placebo group after 4 weeks. Perera et al. [98] compared the effect of ω-3 PUFAs (592,74 mg/day) + ω-6 PUFAs (361.5 mg/day) treatment versus placebo in 94 children with ADHD taking MPH. They reported significant improvement in the active group compared to placebo after six months in symptoms of restlessness, inattention and impulsiveness, but they did not measure them with standardized tools.

Finally, Raz et al. [96] and Dubnov-Raz et al. [95] supplemented children with short chain fatty acids (respectively, ALA or a combination of higher LA and ALA) and they both reported no significant beneficial effect for any of the variables examined.

## 3. Conclusions and Future Directions

The aim of this review was to provide a comprehensive overview of the effects of ω-3 PUFAs in three major neuropsychiatric disorders: psychosis, ASD, and ADHD (Table 2). The interest on these illnesses raised from the evidence suggesting that the onset of these disorders arise during the human brain development in which ω-3 PUFAs play a central role [100]. Indeed, it has been reported that DHA exerts several functions in process of neurogenesis, neurotransmission and protection against oxidative stress [101,102] and it starts to accumulate in brain during pregnancy, especially in the second half of gestation [103,104,105], coinciding with the growth spurt in the grey matter [105]. Several theories have been proposed to explain why deficits and imbalances of ω-3 PUFAs can be linked to impairments in cognitive and behavioral performances [101,106]. Therefore, in the last decade, we found a growing number of randomized controlled trials (RCT) testing the efficacy of ω-3 PUFAs alone or as add-on therapy in the treatment of different psychiatric disorders. However, the available evidence is not always in agreement, probably due to the heterogeneity of the methods employed by the original studies, which often had small and not homogeneous sample size, different selection criteria, different subtypes and dosage of ω-3 PUFAs (e.g., EPA, DHA, a combination of the two, or the addition of omega-6 PUFAs) as well as various duration of supplementation. Another relevant limiting methodological point is represented by the multitask nature of the neuropsychological tests and scales, leading to results unavoidably biased without adjusting for multiple tests—and in this case, a significant result may hardly exist.

Despite these limitations, overall, the results seem to highlight the beneficial role of ω-3 PUFAs in the three neuropsychiatric disorders described above. Specifically, for psychotic patients, it has been reported that ω-3 PUFAs supplementation could be effective in preventing transition to psychosis and in the treatment of severity symptoms and global functioning.

Furthermore, the available evidence also suggests that the detection and treatment of ω-3 PUFAs deficiency early in the course of illness may be required to exert the greatest protection against transition to psychosis and severity of symptoms. However, this hypothesis requires future investigations.

With regard to ADHD, the effect of ω-3 PUFAs supplementation has been widely studied in this disorder via RCTs. However, also in this case, the great methodological heterogeneity across studies, including variations in sample size, study duration, type and dosage of supplementation, makes difficult to compare the findings and draw firm conclusions about the efficacy. We found 13 out of 25 studies reporting some beneficial effect of ω-3 PUFAs on ADHD symptoms, whereas the other 12 found negative results. A common characteristic shared by RCTs reporting positive outcomes is that they all have—in tandem or alone—a minimum sample size of 50 and minimum study duration of 15 weeks. In terms of supplementation, they involve both EPA and DHA, except for two studies whose ω-3 PUFAs composition is not declared. Studies that add GLA to EPA and DHA were more likely to obtain positive than negative outcomes. On the other hand, looking at studies reporting negative outcomes, they follow a different methodology compared to positive studies, including the use of small sample sizes, short study duration (less than 15 weeks), supplementations with either DHA or EPA alone, the lack of a placebo arm. An exception is represented by Milte and coworkers’ study [84], which has sample size, type of supplementation and study duration comparable to that of other studies with positive results, but failed to find a significant treatment effect of EPA or DHA over placebo. Authors attributed the negative finding to the sample size, considered too small compared with that required to provide sufficient power to detect a significant treatment effect.

Moreover, considering the eight studies exploring the effect of ω-3 PUFAs as an add-on therapy to MPH or ATX, six failed to find a better outcome for combined treatment versus pharmacotherapy alone, whereas only two observed a greater improvement in the combined treatment group. However, as described above, negative studies—more than positive ones—have several weaknesses that could have affected the results so that it is still premature to draw conclusions about the appropriateness of combined treatment for ADHD. Interestingly, Barragán et al. [89], despite finding no increased benefit on symptoms of combined ω-3/ω-6 PUFAs and MPH over MPH monotherapy, observed that children receiving combined treatment needed lower doses of MPH to achieve the same clinical improvement of those receiving MPH alone and had a lower rate of withdrawal and a lower incidence of adverse events.

What has frequently emerged from recent meta-analyses [29,107,108,109] is a small but significant effect of ω-3 PUFAs in ADHD composite symptoms that is considered, on one hand, too modest to recommend ω-3 PUFAs in lieu of existing pharmacotherapies, whereas on the other hand, enough reliable to justify the use of ω-3 PUFAs as coadjutors of pharmacologic treatments, given their better tolerability in terms of side effects. More high-quality RCTs are encouraged in order to clarify the effectiveness of ω-3 PUFAs supplementation in children and adolescents with ADHD. Future studies should use primarily both EPA and DHA within double-blind designs, recruit samples of adequate sizes and provide supplementation for at least six months [109] to support fatty acid turnover in the brain, which is thought to be quite low especially in 6–12-year-old children [110]. Hopefully, more reliable investigation techniques are welcome to prevent the dispersion typical of multitask scales and multiple tests, whose use leads to the unavoidable loss of statistical power, whichever the study design and sample size.

Finally, regarding ω-3 PUFAs and ASD, the limited evidence available is insufficient for drawing any conclusion. The trials included in this review varied in their durations, sample size and amounts of ω-3 PUFAs supplementation, therefore there is still uncertainty about the effects of these fatty acids on symptoms of ASD in children. In particular the three RCTs included have different duration (six weeks, six months and 16 weeks), while ω-3 PUFAs erythrocyte membranes may reach a steady state after six months and at least four months is needed to demonstrate an effect on cognitive performance. Even longer study periods of one year might be needed to demonstrate behavioral changes in response to ω-3 PUFAs supplementation [70].

Given the importance of DHA in brain function and development, and its possible implications in the modulation of ASD symptoms, it is important to continue to investigate the positive effects of the supplementation. In this population, the dietary intake of ω-3 PUFAs rich foods is low due to a monotonous dietary pattern: the incorporation of DHA into cellular membranes may be therefore insufficient.

In conclusion, the lack of consistency across studies that have explored the ω-3 PUFAs effects in these major neurodevelopmental disorders implies the necessity of larger prospective interventional clinical studies, with intervention starting at the beginning of illness, utilizing therapeutic dosage of ω-3 PUFAs, and new methodologies of investigation. Despite all the uncertainties that have been mentioned, we do believe that this is an exciting area for future scientific studies since it will help elucidate the critical role played by ω-3 PUFAs in human brain development with wide-reaching implications in health and disease.

## 4. Material and Methods

A comprehensive search on PUBMED of all RCTs using ω-3 PUFAs on patients with autism, ADHD and psychotic symptoms published up to August 2017 was performed. Articles of potential interest were identified by using the following search terms: “omega-3“, ”polyunsaturated fatty acids”, “PUFAs”, “trial”, “EPA”, “DHA”, “docosahexaenoic acids”, “eicosapentaenoic acids” combined with the following term: “autism”, “neurodevelopmental spectrum disorder”, “attention deficit hyperactivity disorder”, “adhd”, “psychosis”, “psychotic disorder” or “psychotic symptom” or “first psychotic episode”, “ultrarisk psychosis”, “risk psychosis”. In this review, RCTs examining the efficacy of ω-3 PUFAs in young adults, adolescents and children with autism, ADHD and psychotic symptoms were selected. Trials were included if they examined the efficacy of ω-3 PUFAs to target autism, ADHD and psychotic symptoms as a primary outcome and in case they investigated the efficacy of ω-3 PUFAs in transition to psychosis status in the context of subjects with risk or ultra-risk for psychotic disorder. We included also subjects at risk for ADHD if the authors used validated rating scales to measure the presence and severity of ADHD symptoms as a primary outcome. We considered only trials in which the authors used an exposure of ω-3 PUFAs as a unique treatment or as an adjunctive therapy to other drugs (e.g., antipsychotics, stimulants), or other no pharmacological strategies such as psychotherapy, vitamin and mineral supplements compared to placebo or pharmacotherapy alone.

**Figure 1:**
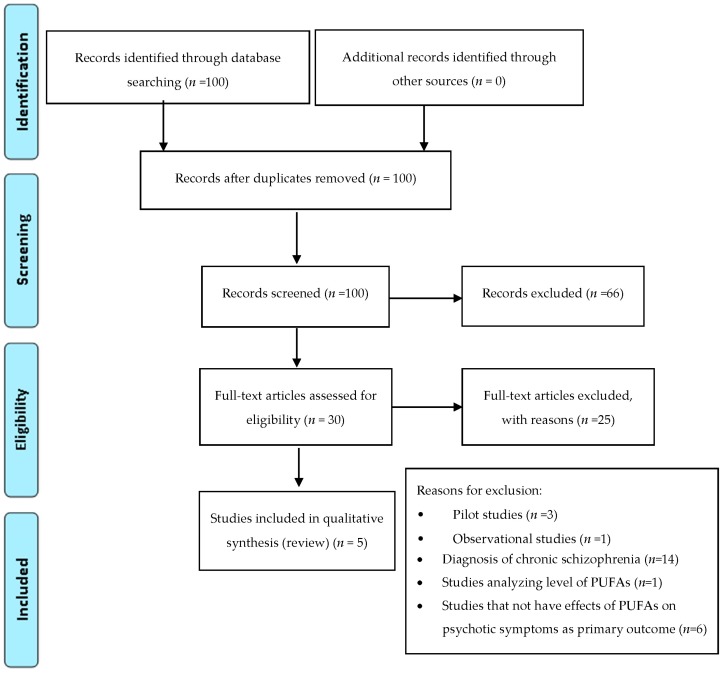
PRISMA diagram: ω-3 PUFAs supplementation in early psychotic symptoms.

**Figure 2:**
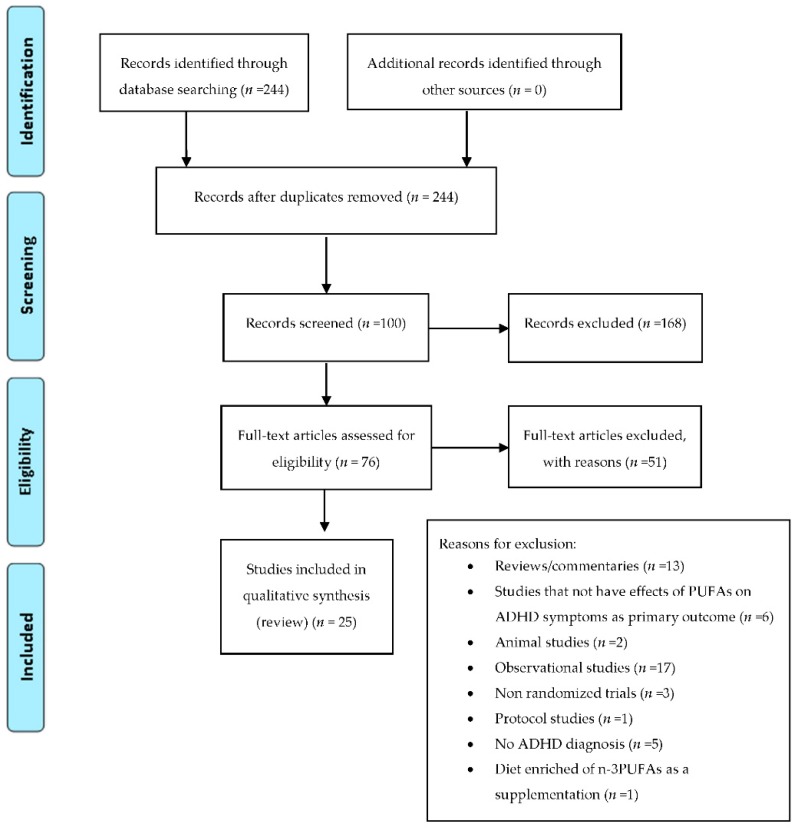
PRISMA diagram: ω-3 PUFAs supplementation in ADHD.

**Figure 3:**
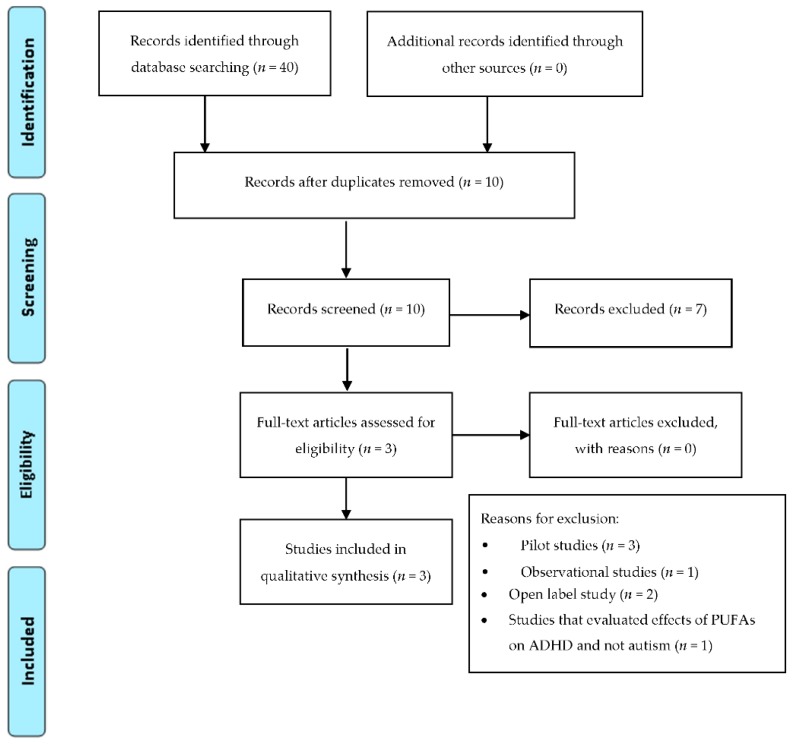
PRISMA diagram: ω-3 PUFAs supplementation in ASD.

To limit the heterogeneity of this review and to reduce selection biases, we decided to exclude: trials examining the efficacy of ω-3 PUFAs in treating psychotic symptoms in subjects with diagnosis of chronic schizophrenia; trials examining the efficacy of ω-3 PUFAs in treating ADHD symptoms in subjects with diagnoses different from ADHD (e.g., dyslexia, developmental coordination disorder); trials involving healthy subjects; trials analyzing levels of ω-3 PUFAs; studies that not explore the effects of ω-3 PUFAs on autism, ADHD and psychotic symptoms as primary outcome. In addition, we excluded trials that employed diet enriched of ω-3 PUFAs as a supplementation. Among the 384 articles retrieved, 116 RCT studies were identified and screened by reading the abstract and, when necessary, the full text, in order to select those articles relevant for the analysis. A manual search of bibliographic cross-referencing complemented the search. Reference lists of relevant papers were also inspected to identify any additional trials. Relevant articles were obtained and included in the review if they used an exposure of ω-3 PUFAs, included autism, ADHD and psychotic symptoms as an outcome measure, enrolled human participants, included a comparison group, and reported a trial. The process of identification and inclusion of trials is summarized in Figure 1, Figure 2 and Figure 3. Finally, 33 trials were included for the review. All searches, trial identification, data abstraction, and tabulation were completed independently by 2 researchers. Discordances were discussed and resolved.

## Figures and Tables

**Table 1:** RCTs of ω-3 PUFAs supplementation in early psychotic symptoms (ultra-high risk of psychosis and first psychotic episode), ADHD and ASD.

**Ultra Hight Risk Psychosis**					
**Study**	**N. Sample**	**N-PUFA Assessed Daily Amounts**	**Duration (Weeks)**	**Outcome Measures**	**Major Finding**
[46]	76 of 81 participants (93.8%) completed the intervention (aged 13–25)	MONO 1.2 g/day (EPA 700 mg + DHA 480 mg) or PLACEBO + 7.6 mg Vit E	12 + 40 of follow up	Primary outcome transition to psychosis Secondary outcomes included symptomatic and functional changes	A significant reduction in ω-3 PUFAs group of positive symptoms, negative symptoms, and general symptoms and improved functioning compared with placebo.
[51]	15	MONO 1.2 g (EPA + DHA) day OR PLACEBO	12	PANSS, MADRS GAF Udvalg for Kliniske Undersøgelser (SCALE for SIDE EFFECTS)	ω-3 PUFA significantly improved functioning and reduced psychiatric symptoms, compared with placebo. Side effects did not differ between the treatment groups
[50]	304 (aged between 13 and 40 years)	MONO 1.4 g (EPA + DHA) or PLACEBO + CBCM	24	Transition to psychosis status at 6 months BPRS SANS MADRS YMRS SOFAS GF: S GF: R	ω-3 PUFAs are not effective under conditions where good quality, evidence-based psychosocial treatment is available
**First episode Psychosis**					
**Study**	**N. Sample**	**N-PUFA Assessed Daily Amounts**	**Duration (Weeks)**	**Outcome Measures**	**Major Finding**
[55]	ADD ON 2 g EPA OR PLACEBO	80 FEP but only 69 were eligible for analysis	12	Primary outcome PANSS GAF CGI Secondary outcome measures: tolerability measures and cumulative anti psychotic dose	EPA no efficacy on specific psychotic symptoms
[54]	71 (aged 16–35)	ADD ON 2.2 g/day (EPA + DHA) or PLACEBO	26	PANSS	Significant differences between the study arms regarding total PANSS score change favoring ω-3 PUFA.
**ADHD**					
**Study**	**N. Sample**	**N-PUFA Assessed Daily Amounts**	**Duration (Weeks)**	**Outcome Measures**	**Major Finding**
[75]	50	ADD ON ATX + 180 mg EPA + 120 mg DHA OR ATX	16	KSAD S-PLCPRS	Study group had greater but not statistically significant (*p* = 0.08) reduction in CPRS ADHD scores.
[88]	40	ADD ON MPH + 241 mg DHA + 33 mg EPA + 180 mg omega 6 per day OR MPH + placebo	10	ADHD-RS Side-Effect questionnaire	ADHD-RS scores decreased significantly within both groups but total score and scores of inattention, hyperactivity, and impulsivity were not significantly different between the groups.
[89]	90	ADD ON 558 mg EPA + 174 mg DHA + 60 mg GLA per day OR MPH + 558 mg EPA + 174 mg DHA + 60 mg GLA per day OR MPH	48	ADHD-RS CGI-S	No significant differences between MPH + Omega-3/6 and MPH alone. Significant differences between Omega-3/6 and MPH + Omega-3/6 in ADHD Total score and Hyperactivity-Impulsivity indicating a stronger effect for combined treatment compared with Omega-3/6.
[78]	69	ADD ON MPH + 240 mg DHA + 360 mg EPA per day OR MPH + placebo	8	ADHD-RS	Both groups had a significant improvement of ADHD symptoms but there was no significant difference between them.
[79]	40	MONO 650 mg DHA + 650 mg EPA per day OR placebo (margarine)	16	fMRI scan with a Go-No-Go task; CBCL; SWAN	Scores on CBCL attention problems were reduced in comparison with placebo (*p* < 0.001). No effect of EPA/DHA on Go-No-Go task or on fMRI measures.
[80]	26	MONO 20–25 mg/kg EPA + 8.5 − 10.5 mg/kg DHA per day OR placebo (sunflower oil)	8	SWAN CPRS-L CPT Go-No-Go test	CPRS subscales measuring symptoms of inattention and impulsivity showed a significant improvement in both groups; study group showed a greater but not statistically significant improvement.
[97]	85	MONO 1 g omega 3 per day OR MPH OR placebo	4	CPRS CTRS KBIT (for IQ)	Significant association between treatment with MPH or omega 3 and improvement in hyperactivity-impulsivity and combined subtypes. No significant association between treatment with placebo and improvement.
[95]	17	MONO 1 g ALA per day OR placebo (lactose)	8	MOXO-CPT CPRS CTRS	No significant differences between groups in the changes from pre- to post-supplementation values on CPRS, CTRS and MOXO-CPT
[81]	92	MONO 500 mg EPA + 2.7 mg DHA and 10 mg Vitamin E per day OR placebo (rape seed oil and medium-chain triglycerides)	15	CPRS CTRS	Supplementation improved CTRS inattention/cognitive subscale (*p* = 0.04) but not Conners’ total score. In oppositional children (*n* = 48) CTRS total score improved ≥25% in 48% of the children in study group vs. 9% for placebo (*p* = 0.001).
[90]	103	ADD ON Ritalin + 635 mg EPA + 195 mg DHA + 100 mg other omega 3 fatty acids per day OR Ritalin + placebo (olive oil)	8	ASQ-P	Significant improvement from week in ASQ-P scores of the omega-3 group.
[76]	64	MONO 558 mg EPA + 174 mg DHA + 60 mg GLA per day OR placebo (olive oil)	12	ADHD-RS CGI-S	Improvement on CGI-S in the study group significantly greater than in the placebo group. More than half of all children did not respond to treatment. However, 26% in the study group responded versus 7% in the placebo group.
[83]	147	MONO 300 mg PS and 120 mg EPA + DHA (EPA/DHA ratio of 2:1) per day OR placebo (cellulose)	15	CPRS CTRS SDQ CHQ-PF50	Significant reduction in the Restless/Impulsive subscale of CPRS and in Parental Emotional Impact subscale of the CHQ in the study group compared to placebo group.
[91]	76	MONO 558 mg EPA + 174 mg DHA + 60 mg GLA + 9.6 mg vitamin E per day OR placebo (medium chain triglycerides)	12	CTRS CPRS BIS	LC-PUFA supplementation did not improve ADHD symptoms at 12 weeks follow-up.
[84]	87	MONO 1109 mg EPA + 108 mg DHA + Vitamine E per day or 264 mg EPA, 1032 mg DHA + Vitamine E per day OR placebo (safflower oil)	48	word reading and spelling subtests from WIAT-III Vocabulary subtest from WISC-III CPRS TEA-ch Go/No-go	No significant treatment effects for literacy, cognition or parent-reported behavior.
[85]	87 ADHD	MONO 1109 mg EPA + 108 mg DHA + Vitamine E per day OR 264 mg EPA + 1032 mg DHA + Vitamine E per day OR placebo (safflower oil)	16	word reading and spelling subtests from WIAT-III Vocabulary subtest from WISC-III CPRS TEA-ch Go/No-go	No significant treatment effects for literacy, cognition or parent-reported behavior.
[98]	94	ADD ON 592.74 mg omega 3 + 361.5 mg omega 6 per day + MPH OR placebo (sunflower oil) + MPH	24	11-item checklist completed by the parents.	Significant improvement in the study group compared with the placebo group (*p* < 0.01) at 12 weeks in restlessness, aggressiveness, completing work and academic performance. Significant improvement became evident at 24 weeks (*p* < 0.05) also for inattention, impulsiveness, and cooperation with parents and teachers.
[96]	63	MONO 480 mg LA + 120 mg ALA + 190 mg mineral oil + 10 mg a-tocopherol per day OR placebo (vitamin c)	7	DSM-IV questionnaire for ADHD Abbreviated CPRS/CTRS TOVA	Both groups ameliorated some of the symptoms, but no significant differences were found between the groups in any of variables explored
[77]	150	ADD ON Ritalin + 100–400 mg EPA per day depending on weight OR Ritalin + 22 mg zinc sulfate OR Ritalin + placebo (sugar)	8	CPRS CTRS	A significant decreasing trend in all three groups was observed. No significant differences between groups on average Conners’ scores at Week 8.
[92]	50	MONO 480 mg DHA + 80 mg EPA + 40 mg AA + 96 mg GLA per day OR placebo (olive oil)	16	ASQ-Parents/Teachers DBD-Parents/Teachers CPT WJ-R	ASQ: Significant decrease from baseline within both placebo and PUFA group but no treatment effect. DBD: For conduct problems rated by parents and for attention symptoms rated by teachers, study group showed significant improvement over the placebo group.
[86]	60	MONO 156 mg EPA + 95 mg DHA containing 300 mg PL-omega 3 per day OR 153 mg EPA + 96 mg DHA containing FO OR placebo (rapeseed oil)	12	TOVA ASQ CBCL 4–18	Total TOVA score was significantly affected by the treatments: PL-omega 3 > FO > placebo. The proportion of subjects with normative scores in the PL-omega-3 group (11/18) was statistically different from that of the placebo group (3/21), but not from the FO group (7/21). The PL-omega-3 and, to a limited extent, the FO group significantly improved the executive functioning in almost all TOVA-adjusted variables compared with placebo
[99]	54	ADD ON MPH + 345 mg DHA per day OR MPH + not specified placebo	16	TOVA Children’s Color Trails Test CBCL CPRS	No statistically significant improvement in any measure of ADHD symptoms.
[87]	95	MONO 600 mg EPA + 120 mg DHA + 15 mg of vitamin E per day OR Placebo (olive oil)	16	DISYPS-II CBCL TRF HAWIK IV KITAP 6–10 years TAP 10–18 years	Omega-3 improved working memory function, but had no effect on other cognitive measures and on DISYPS-II, CBCL or TRF.
**Elevated ADHD Symptoms**					
**Study**	**N. Sample**	**N-PUFA Assessed Daily Amounts**	**Duration (Weeks)**	**Outcome Measures**	**Major Finding**
[82]	112	MONO 21.9/29.2 mg EPA + 16.5/22 mg DHA + 300/400 mg natural mono-unsaturated olive oil + 0.675/0.9 mg vitamin E per day depending on age OR placebo (olive oil, lecithin, coconut oil and β-carotene)	14	CPRS COMPASS cognitive battery TOVA BRUMS EEG CGI	No significant difference between treatment groups on CPRS. Significant treatment effects on CPRS scores of hyperactivity, attention, learning and probability of ADHD for children who did not meet criteria for combined hyperactivity and inattention. Significant improvements in the study group on delayed working memory between baseline and Week 8.
[93]	104	MONO 558 mg EPA + 174 mg DHA+ 60 mg GLA + 10.8 mg vitamin E per day, alone or associated with a multivitamin/mineral supplement OR placebo (palm oil)	15	CPRS CTRS	CPRS: Significant improvements in the PUFA groups (combined) compared with placebo on core ADHD symptoms (inattention, hyperactivity, and impulsivity) and on ratings of oppositional behavior CTRS: No significant results.
[94]	109	MONO 558 mg EPA + 174 mg DHA+ 60 mg GLA + 10.8 mg vitamin E per day, alone or associated with a multivitamin/mineral supplement OR placebo (palm oil)	15	CPRS CTRS Creature-Counting test Short form of WISC-III Inspection time Rey Auditory-Verbal Learning Test Knock and Tap NEPSY subtest Stroop color–word test	Creature Counting: Over 15 weeks, there was a significant improvement in the PUFA groups compared to placebo.
**Autism**					
**Study**	**N. Sample**	**N-PUFA Assessed Daily Amounts**	**Duration (Weeks)**	**Outcome Measures**	**Major Finding**
[71]	57	1.3 g of ω-3 PUFA per day OR placebo	6	the change in hyperactivity on the Aberrant Behavior Checklist (ABC-H, parent and teacher) Secondary outcomes included changes in the other subscales of the ABC and the Social Responsiveness Scale (SRS).	Children in the omega-3 fatty acid group had a greater reduction in hyperactivity, but the difference was not statistically significant. This study found statistically significant improvements in the ω-3 PUFA group in the stereotypy and lethargy subscales of the ABC.
[72]	48	DHA supplementation of 200 mg/day OR placebo.	26	The primary outcome measure was a significant positive response on the Clinical Global Impressions-Improvement (CGI-I) scale. Secondary outcome measures were changes in behavior or development noted on the Aberrant Behavior Checklist (ABC), the Behavior Assessment Scale for Children (BASC), and the Child Development Inventory (CDI)	The DHA group was not rated as improved in core symptoms of autism compared to the placebo group on the CGI-I. Parents (but not teachers) provided a higher average rating of social skills on the BASC for the children in the placebo group compared to the DHA group Teachers (but not parents) provided a higher average rating of functional communication on the BASC for the children in the DHA group compared to the placebo group
[73]	13	240 mg of ARA-enriched triglyceride (40 mg/capsule each of ARA and DHA, and 0.16 mg/capsule of astaxanthin). The usual daily doses of this capsule were 6 capsules (240 mg). The placebo was an identical capsule containing olive oil.	16	The outcome measures were the Social Responsiveness Scale and the Aberrant Behavior Checklist-Community.	This supplementation regimen significantly improved Aberrant Behavior Checklist-Community-measured social withdrawal and Social Responsiveness Scale-measured communication.

**Table 2:** Effects of ω-3 PUFAs supplementation in psychosis, ADHD, ASD: summary.

Diagnosis	Positive Results	Negative Results	Positive Results Without Statistical Significance
Ultra Hight Risk Psychosis	[46,51]	[50]	
First psychosis episode	[54]	[55]	
ADHD	[76,79,81,83,86,90,98]	[84,85,87,91,95,99]	[75,77,78,80,88,89,92,96,97]
Elevated ADHD symptoms	[93,94]		[82]
Autism	[73]	[72]	[71]
